# Isolation and characterization of genes functionally involved in ovarian development of the giant tiger shrimp *Penaeus monodon* by suppression subtractive hybridization (SSH)

**DOI:** 10.1590/s1415-47572010000400014

**Published:** 2010-12-01

**Authors:** Rachanimuk Preechaphol, Sirawut Klinbunga, Bavornlak Khamnamtong, Piamsak Menasveta

**Affiliations:** 1 Program in Biotechnology, Faculty of Science, Chulalongkorn University, BangkokThailand; 2 Center of Excellence for Marine Biotechnology, Faculty of Science, Chulalongkorn University, BangkokThailand; 3 Aquatic Molecular Genetics and Biotechnology Laboratory, National Center for Genetic Engineering and Biotechnology, National Science and Technology Development Agency, PathumthaniThailand; 4 Department of Marine Science, Faculty of Science, Chulalongkorn University, BangkokThailand

**Keywords:** EST, SSH, *Penaeus monodon*, ovarian development, semiquantitative RT-PCR

## Abstract

Suppression subtractive hybridization (SSH) libraries between cDNA in stages I (previtellogenic) and III (cortical rod) ovaries of the giant tiger shrimp (*Penaeus monodon*) were established. In all, 452 ESTs were unidirectionally sequenced. Sequence assembly generated 28 contigs and 201 singletons, 109 of which (48.0%) corresponding to known sequences previously deposited in GenBank. Several reproduction-related transcripts were identified. The full-length cDNA of *anaphase promoting complex subunit 11* (*PmAPC11*; 600 bp with an ORF of 255 bp corresponding to a polypeptide of 84 amino acids) and *selenoprotein Mprecursor* (*PmSePM*; 904 bp with an ORF of 396 bp corresponding to a polypeptide of 131 amino acids) were characterized and reported for the first time in penaeid shrimp. Semiquantitative RT-PCR revealed that the expression levels of *PmSePM* and *keratinocyte-associated protein 2* significantly diminished throughout ovarian development, whereas *Ser/Thrcheckpoint kinase 1* (*Chk1*), *DNA replication licensing factor mcm2* and *egalitarian* were down-regulated in mature ovaries of wild *P. monodon* (p < 0.05). Accordingly, the expression profiles of *PmSePM* and *keratinocyte-associated protein 2* could be used as biomarkers for evaluating the degree of reproductive maturation in domesticated *P. monodon*.

## Introduction

The giant tiger shrimp (*Penaeus monodon*) is one of the most economically important cultured species (Bailey-Brock and Moss, 1992; Rosenberry, 2001). Breeding *P. monodon* in captivity, besides being difficult (Withyachumnarnkul *et al.*, 1998; Wongprasert *et al.*, 2006), is very much restricted by the current dependency on wild-caught broodstock, with the consequential overexploitation of high-quality sources in the wild. As a result, aquacultural production of *P. monodon* has undergone a significant decline over the last several years (Limsuwan, 2004).

The low degree of reproductive maturation of captive *P. monodon* has also limited the ability to genetically improve this important species by domestication and selective breeding programs (Withyachumnarnkul *et al.*, 1998; Kenway *et al.*, 2006; Preechaphol *et al.*, 2007). Eyestalk ablation is used commercially to induce ovarian maturation in penaeid shrimp but the technique leads to an eventual loss in egg quality and death of the spawners (Benzie, 1998). Therefore, predictable maturation and spawning of captive penaeid shrimp without the use of eyestalk ablation is a long-term goal for the industry (Quackenbush, 1992).

Basic information on ovarian development is somewhat limited in this shrimp. Initial steps towards an understanding of the molecular mechanisms involved in ovarian and oocyte development in this economically important species, are the identification and characterization of genes differentially expressed in the diverse stages of the process (Preechaphol *et al.*, 2007).

Recently, genes expressed in the shrimp's vitellogenic ovaries were identified and characterized. A total of 1051 clones from a conventional cDNA library were unidirectionally sequenced from the 5' terminus. The nucleotide sequences of 743 EST (70.7%) significantly matched known genes previously deposited in GenBank (*E*-value < 10^-4^), whereas 308 ESTs (29.3%) were regarded as newly unidentified transcripts (*E*-value > 10^-4^). A total of 559 transcripts (87 contigs and 472 singletons) were obtained after sequence assembly. Several reproduction-related genes, viz., *chromobox protein*, *ovarian lipoprotein receptor*, *progestin membrane receptor component 1* and *ubiquitin-specific proteinase 9, X chromosome*, were isolated and characterized (Preechaphol *et al.*, 2007).

Suppression subtractive hybridization (SSH) is widely used for isolating differentially expressed genes in any two closely related samples, specimens or species (Diatchenko *et al.*, 1996). This technique should facilitate the identification of genes involved in ovarian (and oocyte) development. The genes identified could further assist in the domestication and selective breeding programs of *P. monodon.*

In order to provide a further insight into the molecular mechanisms involved in the reproductive maturation processes of *P. monodon*, we carried out SSH of genes expressed in stages I and III ovaries of wild *P. monodon*. The expression profiles of five reproduction-related genes during ovarian development in wild *P. monodon* broodstock were further examined using semiquantitative RT-PCR. Candidate biomarkers for evaluating the degrees of reproductive maturation in captive shrimp are reported herein.

## Materials and Methods

###  Experimental animals

Four-month-old juveniles of *P. monodon*, with body weights of approximately 25-30 g, were purchased from a commercial farm in Chachoengsao (eastern Thailand). These were cultured in 15 ppt seawater at ambient temperature (28-32 °C) and a natural daylight cycle. Broodstock shrimp, with body weights of > 200 g, were wild-caught from Satun, located in the Andaman Sea, west of peninsular Thailand. Prior to SSH library construction, ovaries were dissected out from two broodstock and weighed. The gonadosomatic index (GSI), *i.e.*, ovarian weight/body weight x 100, of each shrimp was calculated. In order to determine expression profiles of reproduction-related genes during *P. monodon* ovarian development, female juveniles and the broodstock were acclimated at normal farm conditions (28-30 °C, natural daylight and 35 ppt seawater) for 2-3 days. Ovarian developmental stages of broodstock were classified according to GSI: < 1.5, 2-4, > 4-6 and > 6% for previtellogenic (I), vitellogenic (II), early cortical rod (III) and mature (IV) ovaries (*N* = 4 for each stage), respectively. Ovaries were dissected from each shrimp immediately after collection and kept at -80 °C until use.

###  Isolation of total RNA and mRNA

Total RNA was extracted from various tissues of each individual with TRI-Reagent (Molecular Research Center) and mRNA was further purified using a QuickPrep Micro mRNA Purification Kit (GE Healthcare). Total RNA and mRNA were kept under absolute ethanol at -80 °C, prior to reverse transcription.

###  Construction of suppression subtractive hybridization (SSH) cDNA libraries and EST analysis

Initially, two micrograms of mRNA from the ovaries of the *P. monodon* broodstock were reverse-transcribed. Suppression subtractive hybridization (SSH) between cDNA from stages III (GSI = 5.69%) and I (1.43%) and vice versa (Diatchenko *et al.*, 1996) was carried out using a PCR-Select cDNA Subtraction Kit (BD Clontech). The subsequent products were ligated to pGEM-T Easy vector and transformed into *E. coli* JM109. Plasmid DNA was extracted from clones carrying > 300 bp inserts and unidirectionally sequenced using the M13 reverse primer. Sequencing data were pre-processed to remove low-quality sequences (sequence length < 100 bp, the percentage of undetermined bases > 3% and low complexity), by using SeqClean with option-A to disable the trimming of poly A tail. Repetitive sequences matching the RepBase dataset were masked by using RepeatMasker. Sequence clustering and assembly was done using TIGR Gene-Indices Clustering Tools (TGICL) (Pertea *et al.*, 2003) with CAP3 (Huang and Madan, 1999). Nucleotide sequences of assembled and non-assembled ESTs were compared with GenBank data using BlastN and BlastX (Altschul *et al.*, 1990). Significantly matches to nucleotides/proteins were considered when the *E*-value was < 1 x 10^-4^. Blast2GO was used for the additional annotation of biological activities in BlastX matched sequences, thereby enabling Gene Ontology (GO) prediction of sequence data for which no GO annotation is, as yet, available (Conesa *et al.*, 2005).

ESTs representing *P. monodonselenoprotein M precursor* (*PmSePM*) and *anaphase promoting complex subunit 11* (*PmAPC11*) were further sequenced from the reverse direction of the original cDNA clones by using a M13 forward primer.

###  Semiquantitative RT-PCR

Expression profiles of *keratinocyte-associated protein 2*, *Ser/Thrcheckpoint kinase 1*, *DNA replication licensing factor mcm2*, *PmSePM* and *egalitarian* during ovarian development of *P. monodon* broodstock were analyzed by way of semiquantitative RT-PCR. *EF-1*α was included as the positive control. Initially, nonquantitative RT-PCR (Klinbunga *et al.*, 2009) was carried out using 100 ng of first-strand cDNA as the template, with varying concentrations of primers (0.05, 0.10, 0.15, 0.20, 0.25, 0.30 and 0.40 μM, respectively). Primer sequences are listed in Table 1. Optimal concentrations of MgCl_2_ (1.0, 1.5, 2.0, and 3.0 mM) were further selected using an optimized primer concentration. Finally, RT-PCR of these genes was undertaken with an optimized primer and MgCl_2_ concentrations for 20, 22, 24, 27, 30 and 35 cycles. The number of cycles before the product reached an amplification plateau was chosen.

Semiquantitative RT-PCR was undertaken with 1.5 mM of MgCl_2_ and 0.2 μM of primers for the respective target genes, 0.15 μM of primers for *egalitarian* and 0.10 μM of those for *EF 1-*α, as follows: 94 °C for 3 min followed by appropriate cycles (22, 27, 24, 22 and 24 cycles for the target genes and 22 cycles for *EF 1-*α, respectively) of 94 °C for 30 s, 53 °C for 45 s and 72 °C for 45 s and a final extension at 72 °C for 7 min. The amplicon was electrophoretically analyzed through 1.5% agarose gels, and visualized with a UV transilluminator after ethidium bromide staining (Sambrook and Russell, 2001). The intensities of the targets and *EF-1*α were quantified from the gel photograph using the Quantity One software (BioRad), and relative expression levels of investigated transcripts (intensity of targets/intensity of *EF-1*α) in all experimental groups of *P. monodon* were statistically tested using analysis of variance (ANOVA), followed by the Duncan's new multiple range test. Results were considered significant when p < 0.05. The ovaries from five groups of shrimp (juveniles and stages I, II, III and IV broodstock, *N* = 4 for each group) were assayed for expression analysis.

## Results and Discussion

An understanding of the roles of genes functionally involved in ovarian and oocyte development should ultimately lead to a plausible approach for inducing reproductive maturation in *P. monodon*. In this study, 220 and 232 clones, respectively, from the forward (cDNAs from stage III ovaries as the tester and those from stage I ovaries as the driver; GenBank accession no. GW775090-GW775309) and reverse (cDNAs from stage I ovaries as the tester and those from stage III ovaries as the driver; GenBank accession no. GW775310-GW775541) SSH ovarian libraries of *P. monodon* were unidirectionally sequenced and 136 (61.8%) and 133 (57.3%) ESTs, respectively, significantly matched known sequences in GenBank (*E*-value < 10^-4^, Tables 2  and 3). Homologues of *thrombospondin* (*TSP*; 39 ESTs accounting for 17.7% and 26 ESTs accounting for 11.2% of sequenced clones) and *peritrophin* (39 ESTs, 17.7% and 27 clones, 11.6%) were abundantly represented in both libraries similar to results from analyses of the conventional cDNA library of vitellogenic ovaries of *P. monodon* (79 and 87 clones accounting for 7.5 and 8.3% of clones sequenced, respectively; Preechaphol *et al.*, 2007).

Relatively high percentages of unknown transcripts were found in both the forward and reverse SSH ovarian libraries of *P. monodon* (84 and 99 ESTs accounting for 38.2% and 42.7%, respectively; Tables 2  and 3). The percentage of unknown transcripts in these SSH libraries was greater than that in the conventional ovarian (308/1051 clones, 29.3%; Preechaphol *et al.*, 2007) and testicular (290/889 clones, 32.6%; Leelatanawit *et al.*, 2009) cDNA libraries but lower than those found in the forward (112/178 ESTs, 62.9%) and reverse (87/187 ESTs, 46.5%) SSH testicular libraries of *P. monodon*, respectively (Leelatanawit *et al.*, 2008).

After sequence assembly, 16 contigs (from 97 ESTs) and 123 singletons were obtained for the forward and 14 contigs (from 142 ESTs) and 90 singletons for the reverse SSH libraries, respectively. In all, 229 transcripts (28 contigs from 251 transcripts and 201 singletons, *i.e.*, 44.5%) were obtained when both libraries were analyzed simultaneously, of which 109 significantly matched known genes in GenBank (*E*-value < 10^-4^). Disregarding contigs representing *thrombospondin*/*peritrophin* (8 contigs) and unknown proteins (12 contigs), 8 contigs matched *ribosomal protein S6*, *elongation factor 1-*α, *elongation factor 2*, *calreticulin*, *ficolin*, *selenophosphate synthetase*, *70 kDa heat shock-like protein* and *a hypothetical protein*, *AGAP006171-PA*.

**Figure 1:**
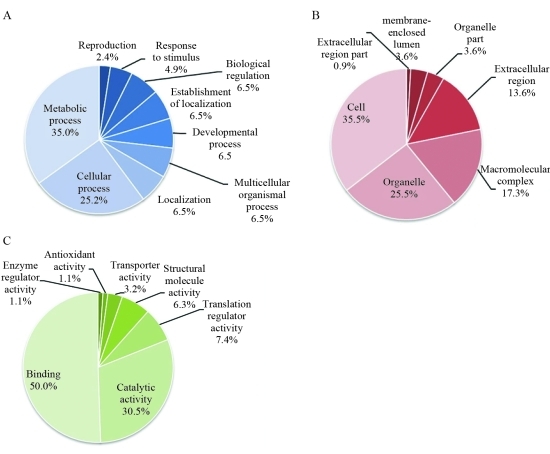
The percent distribution of nucleotide sequences in the SSH ovarian cDNA library of *P. monodon* according to three principal GO categories: A, biological process; B, cellular components and C, molecular functions, respectively.

The percent distribution of nucleotide sequences, according to GO categories of SSH ovarian cDNA libraries of *P. monodon*, was analyzed (Figure 1). In the category `biological process', ESTs involved in metabolic processes were predominant (*e.g.anaphase promoting complex subunit 11*, *S-adenosylmethionine synthetase* and *T-complex protein 1 subunit epsilon*, *i.e.*, 35.0% of the examined ESTs), followed by those involved in cellular processes (*e.g.acidic p0 ribosomal protein*, *DNA replication licensing factor mcm2* and *coatomer protein complex subunit beta*, *i.e.*, 25.2% of the examined ESTs). Reproduction-related ESTs (e.g *RNA binding motif protein 4*, *neuralized protein*, *dynein* and *egalitarian*) were found in 2.4% of the examined sequences of combined SSH data. This discovery rate is higher than that of the conventional ovarian cDNA libraries of *P. monodon* (1.7%; Preechaphol *et al.*, 2007).

As for the category `cellular component', ESTs functionally involved in the cell part (*e.g.myosin II essential light chain*, *ATP synthase E chain* and *Ser/Thr checkpoint kinase 1*, *i.e.*, 35.5% of the examined ESTs) predominated, followed by those functionally displayed in organelles (*e.g.selenoprotein M precursor*, *keratinocyte-associated protein 2* and *interleukin enhancer binding factor 2*; 25.5% of the examined ESTs).

In the category `molecular function', ESTs involved in binding (*e.g.carbonyl reductase*, *translation initiation factor eif-2b*, *RNA binding motif protein 5 isoform 9* and *selenophosphate synthetase*, *i.e.*, 50.5% of the examined ESTs) predominated followed by those displaying catalytic activity (*e.g.MGC80929 protein isoform 1*, *oncoprotein nm23* and *eukaryotic initiation factor 4A*, *i.e.*, 30.5% of the examined ESTs).

The highly organized eukaryotic cilia and flagella contain approximately 250 proteins (Inaba, 2003). They are constructed around evolutionarily conserved microtubule-based structures called axonemes (nine outer doublet microtubules, dynein arms, a central pair of microtubules and radial spokes) (Luck, 1984; Dutcher, 1995; King, 2000). Dynein is functionally related to the transport of various cytoplasmic organelles (Aniento *et al.*, 1993). In *Drosophila*, egalitarian binds to the dynein light chain. Point mutations that specifically inhibit Egl-Dlc association disrupt microtubule-dependant trafficking both to and within the oocyte, thereby resulting in a loss of oocyte fate maintenance and polarity (Carpenter, 1994).

The physiological role of carbonyl reductase was thought to be an NADPH-dependent reduction in a variety of endogenous and foreign carbonyl compounds. However, increasing evidence indicates its involvement in steroid metabolism. In ayu, its localization in ovaries, enzymatic characteristics and transcriptional increase with oocyte maturation, infer its additional function as 20β-HSD in the production of maturation inducing hormones (MIH) (Tanaka *et al.*, 2002).

The DNA replication (or origin) licensing system is prominant in ensuring precise duplication of the genome in each cell cycle, besides being a powerful regulator of metazoan cell proliferation (Eward *et al.*, 2004). The protein kinase Chk1 plays a role in checkpoint control. Recombinant *Xenopus* Chk1 phosphorylates the mitotic inducer Cdc25 *in vitro* at multiple sites. Nevertheless, only XChk1-catalyzed phosphorylation of Cdc25 at Ser-287 is sufficient to confer the binding of 14-3-3 proteins (Kumagai *et al.*, 1998). Moreover, the meiotic maturation of oocytes is regulated by the maturation promoting factor (MPF), a complex of cdc2 (Cdk1), cyclin B and other Cdk/cyclin complexes (Kobayashi *et al.*, 1991; Kishimoto, 1999, 2003). Chk1-dephophorylated Cdc25 activates MPF, thereby causing meiotic resumption in oocytes (Kishimoto, 2003).

Recently, the full length cDNA of *keratinocyte-associated protein 2* was isolated in the Pacific white shrimp (*Litopenaeus vannamei*), although the function of this protein is still unknown. Moreover, its expression was altered following infection by the White Spot Syndrome Virus, WSSV (Clavero-Salas *et al.*, 2007).

The full length cDNAs of *anaphase promoting complex subunit 11* (biological process GO:0008152; GenBank accession no. GW775392) and *selenoprotein M precursor* (cellular component GO:0005783; GenBank accession no. GW775333) were hereby reported and identified for the first time in penaeid shrimp.

**Figure 2:**
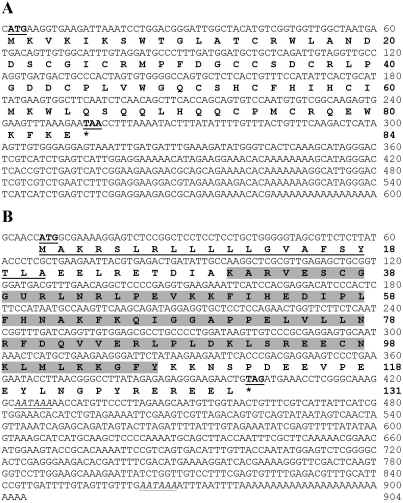
The full length cDNA and deduced protein sequences of *PmAPC11* (600 bp, ORF of 255 bp corresponding to a deduced polypeptide of 84 amino acids; GenBank accession no. GW775392) and *PmSePM* (904 bp, ORF of 396 bp corresponding to a deduced polypeptide of 131 amino acids; GenBank accession no. GW775333). The putative start and stop codons are illustrated in boldface and underlined. The predicted signal peptide and poly A additional signals of *PmSePM* are underlined and italicized and underlined, respectively. The predicted Sep15_SelM domain (positions 31-107) found in the deduced PmSePM protein is highlighted.

The *anaphase promoting complex subunit 11* of *P. monodon* (*PmAPC11*) was 600 bp in length, and consisted of an ORF of 255 bp corresponding to a polypeptide of 84 amino acids, with 5' and 3' UTRs of 1 and 387 bp, respectively (Figure 2A). The closest similar sequence to *PmAPC11* was the *anaphase promoting complex subunit 11 homolog* of *Tribolium castaneum* (*E*-value = 1 x 10^-41^). The predicted molecular mass and p*I* of the deduced *PmAPC11* was 9.84 kDa and 7.99, respectively. Activation of the anaphase-promoting complex (APC) by Cdc20 enables anaphase initiation and exit from mitosis (Kramer *et al.*, 1998; Lorca *et al.*, 1998).

The *selenoprotein M precursor* of *P. monodon* (*PmSePM*) was 904 bp in length, and consisted of an ORF of 396 bp, corresponding to a polypeptide of 131 amino acids, and 5' and 3' UTRs of 6 and 502 bp, respectively (Figure 2B). It significantly matched the *selenoprotein M precursor* of *L. vannamei* (*E*-value = 2 x 10^-58^). The predicted molecular mass and p*I* of the deduced PmSePM protein was 15.10 kDa and 7.75, respectively. PmSePM contained a signal peptide located between A_21_ and E_22_, as well as a Sep15_SelM domain (positions 31-107, *E*-value = 1.9 x 10^-34^) that exerts the thiol-disulphide isomerase activity functionally involved in disulphide bond formation of proteins in the endoplasmic reticulum (ER) (Ferguson *et al.*, 2006).

In addition, the EST representing selenophosphate synthetase, an enzyme involved in selenocysteine biosynthesis, was also identified. In humans, selenium deficiency leads to male infertility and susceptibility to viral infections. More than 20 selenoproteins have been identified in higher eukaryotes (Guimaraes *et al.*, 1996; Rayman, 2000; Korotkov *et al.*, 2002) but their functions in ovarian/oocyte development of *P. monodon* remain unknown. The analysis of baseline information, acquired as part of this study addresses the paucity of data and should provide a better understanding of reproductive maturation in cultured female *P. monodon*.

**Figure 3:**
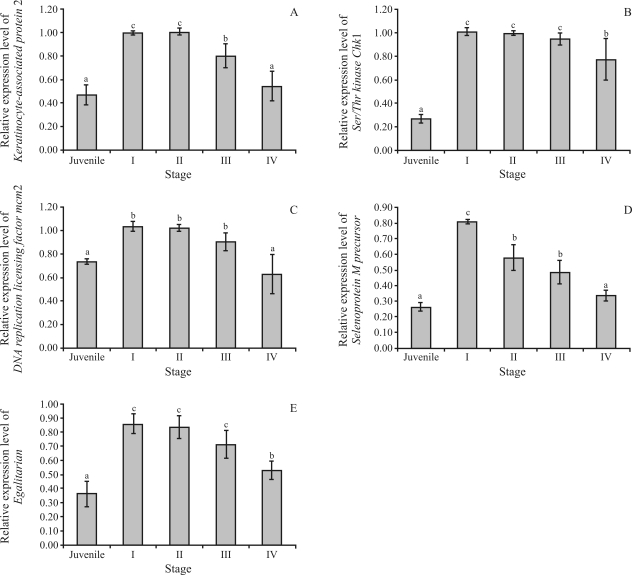
Histograms showing relative expression levels of *keratinocyte-associated protein 2* (A), *Ser/ThrChk1* (B), *DNA replication licensing factor mcm2* (C), *selenoprotein M* precursor (*PmSePM*; D) and *egalitarian* (E) in different ovarian developmental stages of *P. monodon*. For expression analysis, ovaries from 5 groups of shrimp (juveniles and stages I, II, III and IV broodstock, *N* = 4 for each group) were assays. The same letters indicate that the relative expression levels were not significantly different (p > 0.05).

To address the functional involvement of various genes during ovarian development of *P. monodon*, the expression profiles of *keratinocyte-associated protein 2*, *Ser/ThrChk1*, *DNA replication licensing factor mcm2*, *PmSePM* and *egalitarian* were examined by semiquantitative RT-PCR analysis. The control gene (*EF-1*α) seemed to be comparably expressed in all the groups of samples examined, thereby inferring its acceptability for use in normalizing target gene expression. All transcripts were more abundantly expressed in the ovaries of broodstock than juveniles (p < 0.05, Figure 3). The expression level of *PmSePM* peaked in stage I (previtellogenic) of development (GSI < 1.5), to progressively and significantly decrease in stages II (vitellogenic), III (cortical rod) and IV (mature) (p < 0.05). Likewise, *keratinocyte-associated protein 2* was initially down-regulated in stage III, and subsequently, stage IV (p < 0.05). The expression of *Ser/ThrChk1*, *DNA replication licensing factor mcm2* and *egalitarian* during stages I, II and III, was comparable (p < 0.05), although down-regulated in the final stage of ovarian development in wild *P. monodon* broodstock (p < 0.05, Figure 3).

In various animals, a wide variety of maternal mRNA is generally transcribed at the early oogenesis stage, to then be stored in oocytes and carried into fertilized eggs (Qiu *et al.*, 2008; Nishimura *et al.*, 2009). Several reproduction-related genes that are up-regulated during the ovarian development of *P. monodon*, for example, *Ovarian-Specific Transcript 1* (*Pm-OST1*) and *cyclin B* (*PmCyB*), have been previously reported (Klinbunga *et al.*, 2009; Visudtiphole *et al.*, 2009). The down-regulation of *keratinocyte-associated protein 2*, *Ser/ThrChk1*, *DNA replication licensing factor mcm2*, *PmSePM* and *egalitarian* implied that lower levels of these gene products may be necessary for the development and final maturation of *P. monodon* oocytes. The findings facilitate the possible use of RNA interference (RNAi) for studying their functional involvement in *P. monodon* ovarian development. Moreover, the expression profiles of *keratinocyte-associated protein 2* and *selenoprotein M precursor* are potentially applicable as biomarkers to indicate degrees of reproductive maturation in the domesticated shrimp.

In this study, genes expressed in ovaries of *P. monodon* were identified by SSH analysis. The expression profiles of reproduction-related transcripts were examined. Further studies of the molecular mechanisms of those genes and proteins involved in controlling each stage of oocyte maturation should be carried out, to reach a better understanding of the reproductive maturation of *P. monodon* in captivity.

## Figures and Tables

**Table 1:** Nucleotide sequences of primers used for expression analysis of *keratinocyte-associated protein 2*, *Ser/Thrcheckpoint kinase 1*, *DNA replication licensing factor mcm2*, *selenoprotein M precursor* and *egalitarian* in ovaries of wild *P. monodon* broodstock.

Gene	Primer sequence
*Keratinocyte-associated protein 2*	F: 5'-CTGCTGTAAACAATCTGGAAAAC-3'
	R: 5'-GGGACACCTGAGCGGAAGT-3'
*Ser/Thrcheckpoint kinase 1* (*Chk1*)	F: 5'-CTCCCCAGTGTCCTTATTGATTAG-3'
	R: 5'-TGGCTTTCATTCCCTCGCTG-3'
*DNA replication licensing factor mcm2*	F: 5'-TCAAGCGAGACAACAACGAACT-3'
	R: 5'-TTGGACCATCACTGGGCATC-3'
*Selenoprotein M precursor* (*PmSePM*)	F: 5'-GACATCCCACTCTTCCATAAT-3'
	R: 5'-TTTCATCTACAGTTCTTCCCTC-3'
*Egalitarian*	F: 5'-CACTTGTGCCCACTGTCTATG-3'
	R: 5'-CCTCCACTGCCAACACTACTC-3'
*EF-1*α	F: 5'-ATGGTTGTCAACTTTGCCCC-3'
	R: 5'-TTGACCTCCTTGATCACACC-3'

**Table 2:** Examples of known transcripts excluding ribosomal proteins found in the forward ovarian SSH library (cDNAs from stage III ovaries as the tester and those from stage I ovaries as the driver) of *P. monodon*.

Transcript*	Species	Accession number	*E*-value	Size (bp)
*Peritrophin 2*	*Penaeus monodon*	AAM44050.1	5 x 10^-86^	454
*Peritrophin 1*	*Penaeus monodon*	AAM44049.1	4 x 10^-41^	381
*Thrombospondin*	*Penaeus monodon*	AAN17670	1 x 10^-107^	563
*Thrombospondin*	*Marsupenaeus japonicus*	BAC92764.1	3 x 10^-44^	502
*Keratinocyte-associated protein 2*	*Rattus norvegicus*	NP_001099914.1	8 x 10^-25^	470
*Eukaryotic translation initiation factor 2, subunit 2 beta*	*Rattus norvegicus*	AAH62402.1	7 x 10^-11^	605
*Ser/Thr Checkpoint kinase 1 (Chk1), CG17161-PA*	*Drosophila melanogaster*	AAF53552	2 x 10^-22^	417
*Methionyl-tRNA formyltransferase, mitochondrial precursor (MtFMT)*	*Homo sapiens*	NP_640335	4 x 10^-7^	483
*Nucleolin*	*Xenopus laevis*	NP_001081557.1	8 x 10^-4^	380
*Eukaryotic initiation factor eIF-4A*	*Marsupenaeus japonicus*	BAB78485	1 x 10^-41^	279
*26S proteasome regulatory subunit rpn2*	*Culex quinquefasciatus*	XP_001862500	3 x 10^-52^	468
*Cytochrome c oxidase polypeptide IV*	*Bombyx mori*	NP_001073120.1	3 x 10^-38^	405
*Hypothetical protein DKFZp434J1672.1*	*Homo sapiens*	CAB63724	6 x 10^-24^	525
*Coatomer protein complex, subunit beta*	*Gallus gallus*	NP_001006467.1	1 x 10^-67^	588
*Chaperonin containing T-complex polypeptide 1*	*Carassius auratus*	BAA89277	8 x 10^-44^	627
*ATP synthase oligomycin sensitivity conferral protein*	*Toxoptera citricida*	AAU84928	3 x 10^-9^	538
*Cyclin A*	*Asterina pectinifera*	BAA14010	4 x 10^-42^	368
*Non-muscle myosin-II heavy chain*	*Apis mellifera*	XP_393334	8 x 10^-99^	712
*Procollagen-proline, 2-oxoglutarate 4-dioxygenase (protein disulfide isomerase-associated 1)*	*Xenopus tropicalis*	CAJ83276	2 x 10^-47^	663
*Chaperonin containing TCP1, subunit 7*	*Danio rerio*	NP_775355.1	4 x 10^-24^	249
*Isocitrate dehydrogenase 2*	*Tribolium castaneum*	EFA04299	1 x 10^-37^	231
*CD53 antigen*	*Homo sapiens*	NP_001035122.1	4 x 10^-04^	394
*Calreticulin*	*Galleria mellonella*	BAB79277	5 x 10^-103^	714
*DNA replication licensing factor mcm2*	*Xenopus tropicalis*	AAH75567	2 x 10^-47^	490
*RNA binding motif protein 4*	*Aedes aegypti*	XP_001657237.1	6 x 10^-38^	563
*Domino isoform D, CG9696-PD*	*Apis mellifera*	XP_396786	9 x 10^-61^	350
*Eukaryotic translation initiation factor 2B, subunit 5 epsilon, isoform 3*	*Macaca mulatta*	XP_001103944	5 x 10^-32^	713
*Translation initiation factor*	*Anopheles gambiae*	CAD27760.1	2 x 10^-66^	708
*Secreted nidogen domain protein*	*Strongylocentrotus purpuratus*	XP_001196268.1	8 x 10^-09^	466
*Carbamoyl-phosphate synthetase 2, aspartate transcarbamylase, and dihydroorotase*	*Danio rerio*	NP_001009884.1	4 x 10^-18^	611
*DEAD (Asp-Glu-Ala-Asp) box polypeptide 5*	*Tribolium castaneum*	XP_972501.1	3 x 10^-07^	354
*Deleted in malignant brain tumors 1*	*Strongylocentrotus purpuratus*	XP_001180356.1	2 x 10^-04^	486
*ATPase, H+ transporting, lysosomal accessory protein 2, CG8444-PA*	*Tribolium castaneum*	XP_973593.1	1 x 10^-07^	562
*Kinesin-like protein 2*	*Ciona intestinalis*	NP_001011659	5 x 10^-04^	449
*Elongation factor-1 alpha*	*Libinia emarginata*	AAC03149	3 x 10^-102^	713
*Chromosome-associated protein, CG9802-PA, isoform A*	*Apis mellifera*	XP_393700	2 x 10^-74^	652
*CWF19-like 2, cell cycle control*	*Xenopus tropicalis*	NP_001039121.1	1 x 10^-58^	600
*Myosin II essential light chain*	*Tribolium castaneum*	XP_973734	6 x 10^-15^	516
*Gastrula zinc finger protein XLCGF57.1*	*Danio rerio*	XP_001344037.1	4 x 10^-30^	568
*SJCHGC09076 protein*	*Schistosoma japonicum*	AAW26562	6 x 10^-06^	559
*Citrate synthase*	*Aedes aegypti*	EAT45772.1	4 x 10^-75^	478
*Zinc finger protein 146*	*Strongylocentrotus purpuratus*	XP_788425.2	2 x 10^-20^	654
*Sec23 protein*	*Drosophila melanogaster*	NP_730978.1	6 x 10^-63^	465
*Elongation factor-2*	*Libinia emarginata*	AAR01298	8 x 10^-82^	538
*Hypothetical protein TTHERM_00449680*	*Tetrahymena thermophila*	XP_001013363.1	2 x 10^-10^	506
*Calreticulin*	*Bombyx mori*	AAP50845.1	1 x 10^-128^	695
*RNA-binding protein 5*	*Apis mellifera*	XP_394165.3	4 x 10^-43^	713
*Mitochondrial ATP synthase e chain*	*Aedes albopictus*	AAV90734	9 x 10^-16^	403
*Zgc:113377*	*Danio rerio*	NP_001025397	4 x 10^-29^	697
*Inhibitor of Bruton agammaglobulinemai tyrosine kinase*	*Canis familiaris*	XP_539018.2	2 x 10^-12^	634

*Accession no. GW775090-GW775309 for ESTs from the forward SSH library.

**Table 3:** Examples of known transcripts excluding ribosomal proteins found in the reverse ovarian SSH library (cDNAs from stage I ovaries as the tester and those from stage III ovaries as the driver) of *P. monodon.*

Transcript*	Species	Accession number	*E*-value	Size (bp)
*Peritrophin 1*	*Penaeus monodon*	AAM44049.1	2 x 10^-53^	412
*Peritrophin 2*	*Penaeus monodon*	AAM44050.1	1 x 10^-72^	406
*Thrombospondin*	*Penaeus monodon*	AAN17670	3 x 10^-63^	368
*Thrombospondin*	*Marsupenaeus japonicus*	BAC92764.1	9 x 10^-61^	405
*Translation initiation factor eIF4A*	*Spisula solidissima*	AAK85401	1 x 10^-47^	326
*CG10527-like methyltransferase*	*Mesobuthus gibbosus*	CAE53527.1	1 x 10^-28^	458
*Selenoprotein M precursor*	*Homo sapiens*	NP_536355.1	7 x 10^-24^	560
*Stress-70 protein, mitochondrial precursor (75 kDa glucose-regulated protein)*	*Gallus gallus*	NP_001006147.1	1 x 10^-26^	577
*Neuralized protein*	*Drosophila virilis*	AAB60619.1	4 x 10^-27^	575
*Secreted nidogen domain protein*	*Strongylocentrotus purpuratus*	XP_001196268.1	3 x 10^-6^	480
*Thioesterase superfamily member 2*	*Gallus gallus*	XP_419092.1	3 x 10^-13^	511
*Hypothetical protein MGC75603*	*Xenopus tropicalis*	NP_989388	2 x 10^-6^	642
*Carbonyl reductase*	*Plecoglossus altivelis*	BAB92960	2 x 10^-20^	589
*Ovarian lipoprotein receptor*	*Penaeus semisulcatus*	AAL79675	4 x 10^-17^	618
*Allatotropin neuropeptide precursor*	*Spodoptera frugiperda*	CAD98809.1	6 x 10^-9^	402
*Chitin deacetylase-like 9, CG15918-PA*	*Drosophila melanogaster*	NP_611192.1	1 x 10^-17^	353
*Replication factor C/activator 1 subunit*	*Gallus gallus*	AAA20552.1	5 x 10^-58^	583
*Nuclease diphosphate kinase B*	*Danio rerio*	AAF60971	9 x 10^-34^	430
*Acyl-CoA synthase*	*Oceanicola batsensis*	ZP_01000658.1	9 x 10^-51^	518
*70 kD heat shock-like protein*	*Procambarus clarkia*	ABC01063	1 x 10^-103^	692
*Signal sequence receptor*	*Bombyx mori*	NP_001091760.1	3 x 10^-04^	600
*ATP synthase, CG11154-PA isoform A*	*Apis mellifera*	XP_624156	6 x 10^-115^	690
*Ubiquitin-like 1 activating enzyme E1B (SUMO-1 activating enzyme subunit 2)*	*Strongylocentrotus purpuratus*	XP_001195210.1	4 x 10^-24^	473
*Ribonuclease P 40kDa subunit isoform 3*	*Macaca mulatta*	XP_001095772	6 x 10^-19^	688
*Selenophosphate synthetase(selenium donor protein)*	*Drosophila melanogaster*	NP_725374.1	5 x 10^-103^	710
*Peptidylprolyl isomerase D*	*Danio rerio*	NP_001002065.1	1 x 10^-24^	589
*Egalitarian*	*Drosophila melanogaster*	AAF47054.4	3 x 10^-37^	704
*CCR4-NOT transcription complex, subunit 10*	*Tribolium castaneum*	XP_974052	2 x 10^-29^	585
*Protein phosphatase 2c gamma*	*Aedes aegypti*	EAT47444.1	2 x 10^-56^	711
*RNA polymerase I associated factor 53 isoform 1*	*Canis familiaris*	XP_531998	5 x 10^-16^	710
*Splicing factor, arginine/serine-rich 7*	*Apis mellifera*	XP_001122800	2 x 10^-41^	633
*Interleukin enhancer binding factor 2*	*Mus musculus*	NP_080650.1	4 x 10^-31^	332
*Nuclear autoantigenic sperm protein*	*Danio rerio*	NP_956076.1		700
*Cyteine-rich with EGF-like domain 2, CG11377-PA*	*Tribolium castaneum*	XP_971778.1	6 x 10^-25^	510
*Eukaryotic initiation factor 4A*	*Callinectes sapidus*	ABG67961	1 x 10^-64^	569
*ATP lipid-binding protein like protein*	*Marsupenaeus japonicus*	BAB85212	9 x 10^-30^	588
*TRI1, CG7338-PA*	*Apis mellifera*	XP_624169	3 x 10^-41^	708
*Ferritin*	*Litopenaeus vannamei*	AAX55641.1	3 x 10^-31^	306
*Deleted in malignant brain tumors 1*	*Strongylocentrotus purpuratus*	XP_001180356.1	2 x 10^-05^	713
*Transmembrane 4 superfamily member 8 isoform 1/ Tetraspanin 3*	*Homo sapiens*	NP_005715	3 x 10^-10^	596
*Neutral alpha-glucosidase AB precursor (Glucosidase II subunit alpha)*	*Sus scrofa*	NP_999069.1	2 x 10^-49^	707
*Calreticulin precursor (CRP55) (Calregulin)*	*Oryctolagus cuniculus*	NP_001075704.1	4 x 10^-19^	300
*Ataxin1 ubiquitin-like interacting protein*	*Gallus gallus*	NP_001026544	5 x 10^-41^	612
*Hypothetical protein*	*Mus musculus*	XP_922736.3	2 x 10^-15^	403
*HLA-B-associated transcript 3*	*Apis mellifera*	XP_001121013.1	8 x 10^-25^	261
*Cyclin B3, CG5814-PA*	*Apis mellifera*	XP_397108	6 x 10^-46^	427
*Hypothetical protein cgd5_1220*	*Cryptosporidium parvum*	EAK88123.1	2 x 10^-08^	460
*Ring finger protein 2, CG15814-PA, isoform A*	*Tribolium castaneum*	XP_975438.1	9 x 10^-40^	431
*2-Cys thioredoxin peroxidase*	*Aedes aegypti*	AAL37254	1 x 10^-56^	564

*Accession no. GW775310-GW775541 for ESTs from the reverse SSH library.
